# Platelet Endothelial Cell Adhesion Molecule-1 and Oligodendrogenesis: Significance in Alcohol Use Disorders

**DOI:** 10.3390/brainsci7100131

**Published:** 2017-10-16

**Authors:** Chitra D. Mandyam, Emmanuel G. Villalpando, Noah L. Steiner, Leon W. Quach, McKenzie J. Fannon, Sucharita S. Somkuwar

**Affiliations:** 1 VA San Diego Healthcare System, San Diego, CA 92161, USA; egvillal16@gmail.com (E.G.V.); nsteiner@ucsd.edu (N.L.S.); lwquach@ucsd.edu (L.W.Q.); mfannon@vapop.ucsd.edu (M.J.F.); ssomkuwar@vapop.ucsd.edu (S.S.S.); 2 Department of Anesthesiology, University of California San Diego, La Jolla, CA 92161, USA

**Keywords:** alcohol, blood–brain barrier, endothelium, PECAM-1, oligodendroglia, myelin

## Abstract

Alcoholism is a chronic relapsing disorder with few therapeutic strategies that address the core pathophysiology. Brain tissue loss and oxidative damage are key components of alcoholism, such that reversal of these phenomena may help break the addictive cycle in alcohol use disorder (AUD). The current review focuses on platelet endothelial cell adhesion molecule 1 (PECAM-1), a key modulator of the cerebral endothelial integrity and neuroinflammation, and a targetable transmembrane protein whose interaction within AUD has not been well explored. The current review will elaborate on the function of PECAM-1 in physiology and pathology and infer its contribution in AUD neuropathology. Recent research reveals that oligodendrocytes, whose primary function is myelination of neurons in the brain, are a key component in new learning and adaptation to environmental challenges. The current review briefly introduces the role of oligodendrocytes in healthy physiology and neuropathology. Importantly, we will highlight the recent evidence of dysregulation of oligodendrocytes in the context of AUD and then discuss their potential interaction with PECAM-1 on the cerebral endothelium.

## 1. Alcohol Use Disorder: Focus on Pathology Associated with the Disorder

Around 20 million people in the United States alone meet the criteria for alcoholism, diagnosed as moderate-severe alcohol use disorder (AUD) [1,2] and alcohol contributes to more than 85,000 deaths in the country each year [3,4]. Moreover, chronic alcohol use is associated with heart disease, liver problems, and kidney dysfunctions, pulmonary dysfunction including pneumonia and chronic obstructive pulmonary disease [1,5]. At its core, alcoholism is a chronic relapsing disorder associated with loss of control over alcohol intake, leading to escalation of alcohol intake, and the emergence of a negative emotional state when access to alcohol is removed [6]. The addictive cycle includes three stages, namely binge intoxication, withdrawal/negative affect, and preoccupation and anticipation [7]. Neurochemical substrates associated with each stage have been discussed elegantly in the past [6,8], and pharmacological targeting of some of these substrates have been the focus of development of therapeutic strategies for alcoholism [2]. A major challenge to designing pharmacotherapy has been the multigenetic and complex pathophysiology of AUD that includes neuroadaptations in several areas of the brain [8].

Several pathologies associated with alcoholism are closely tied to the production of reactive oxygen species (ROS) and to oxidative stress that results from the catalytic breakdown of alcohol [9]. Several lines of research indicates such oxidative damage leads to cell death that may produce lasting neuroadaptations that contribute to AUD [10,11,12,13]. For example, alcohol toxicity results in a decrease in hippocampal neurogenesis, in frontal cortex cell loss, and in corpus callosum shrinkage [11,14]. The simple endothelial function of glucose uptake was also disrupted by alcohol [15]. Together, these changes may reduce function of critical neurocircuitry in the brain, and therefore, impede learning and memory, impair executive function and decision-making and reduced connectivity between key brain regions, and thereby contribute to the “relapsing” characteristic of AUD. Clinical evidence for glial damage include MRI scans showing a significant atrophy/decreases in white matter in individuals who have a history of chronic alcohol abuse [16]. This atrophy is exacerbated in individuals with alcohol-associated deficiency of thiamine, a natural dietary antioxidant [16]. Converging evidence from clinical and preclinical research overwhelmingly support oxidative stress as a key factor implicated in alcohol-mediated damage that affects neurons, oligodendroglia, microglia, astroglia as well as cerebral vasculature [13,17,18,19,20]. In the context of oxidative damage, alcohol exposure and experience produces glutamate excitotoxicity which is an activator of ROS that occurs in a calcium dependent manner [21,22]. Notably, glutamate excitotoxicity in an alcohol naïve environment produces cerebrovascular endothelial oxidative damage in a glumatatergic NMDA-receptor dependent manner [23,24,25]. However, a direct evidence for glutamate excitotoxicity in alcohol-induced damage of the cerebrovascular endothelium is not apparent, and this effect of alcohol could contribute to alcohol-induced damage of the endothelium and blood-brain barrier disruption [26]. Moreover, the impact of oxidative damage is too expansive to be justified in a single review. Therefore, the current review narrowly focusses on alcohol and withdrawal mediated adaptations specifically in the endothelial system including the blood-brain-barrier and the oligodendroglia.

The blood-brain barrier is a multifaceted endothelial system that protects the sensitive microenvironment of the central nervous system. This function is achieved through the collusion of the several components that comprise the blood-brain barrier, which in vertebrates, consists primarily of specialized unfenestrated endothelial cells supported by the endfeet of perivascular astrocytes, in conjunction with pericytes embedded in the endothelial cell basement membranes [27,28]. The endothelial cells in the blood-brain barrier are connected via tight junctions and are surrounded by a matrix of collagen-IV, laminin, fibronectin, and other matrix proteins [29]. Disruption of blood-brain barrier integrity is a hallmark of numerous pathologies of the nervous system, including Alzheimer’s disease, cerebral ischemia, encephalitis and multiple sclerosis [28,30,31].

The role of the blood-brain barrier in AUD is critical because of two key tenets. (1) Chronic alcohol abuse damages the blood-brain barrier [26], and (2) leaky blood-brain barrier leads to an influx of peripheral factors (cytokines, chemokines, toxins, leukocytes, etc.), that contribute to the gamut of neuronal damage observed in alcohol toxicity [18,20,32]. For example, chronic ethanol exposure in mice models resulted in decreased expression of the tight-junction proteins such as zona occludin-1 and claudin-5 [15,33], thereby compromising the blood-brain barrier. This impaired vascular endothelial integrity enables enhanced infiltration of leukocytes into the brain, leading to subsequent release of more cytokines and proinflammatory agents that further contribute to a pathological inflammatory phenotype in alcoholism [34]. The current review attempts to explain these blood-brain barrier changes from the perspective of the platelet endothelial cell adhesion molecule 1 (PECAM-1), which is a key component of the endothelial cells. For example, PECAM-1 has been implicated in several other neuropathologies that involve blood-brain barrier damage [28,30,31]. PECAM-1 is essential in regulating endothelial cell integrity, especially during an inflammatory challenge [30,35,36,37] and has been identified and utilized as a marker of blood-brain barrier integrity [30]. The most important reason for pursuing PECAM-1 in alcoholism is that PECAM-1 has been shown to mediate enhanced transendothelial migration of monocytes in response to oxidative stress [38]. Since oxidative stress is widely implicated in alcohol toxicity and in AUD [13,17,19,34], it can be hypothesized that PECAM-1 is involved in pathophysiology of AUD.

## 2. Platelet Endothelial Cell Adhesion Molecule-1: Source and Function

### 2.1. Source of PECAM-1

**Figure 1:**
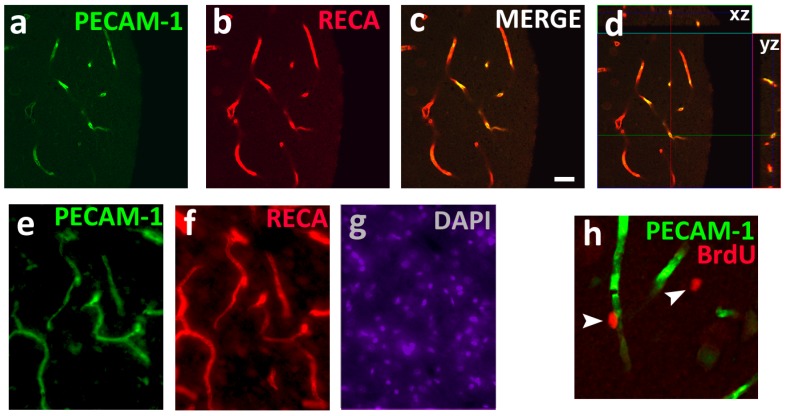
(**a**–**g**) Photomicrographs of prefrontal cortex tissue from adult male Wistar rats stained for platelet endothelial cell adhesion molecule 1 (PECAM-1); 1:500 rabbit anti-PECAM-1; cyanine 2 (CY2), rat endothelial cell antigen (RECA); 1:500 mouse anti-RECA; cyanine 3 (CY3) and 4',6-diamidino-2-phenylindole (DAPI); 1:2000). (**a**–**d**) Single slice confocal images of PECAM-1 (**a**), RECA (**b**) and merge (**c**); z-scan of the colabeling showing an orthogonal view along the xz and yz axis in (**d**). The orthogonal view demonstrates equal penetration of both the antibodies PECAM-1 and RECA, and confirms colabeling (MERGE) of PECAM-1 and RECA shown in panel (**c**). Scale bar in (**c**) is 20 μm, applies (**a**–**g**). (**e**–**g**) Epifluorescent images of PECAM-1 (**e**), RECA (**f**) and DAPI (**g**). (**h**) Confocal image of colabeling of PECAM-1 (CY2) and 28-day-old 5-Bromo-2´-Deoxyuridine (BrdU) cell (1:500 sheep anti-BrdU; CY3) in the prefrontal cortex of the adult male Wistar rat. Scale bar in (**c**) is 30um in (**h**). Arrows point to BrdU cells. Panel (**h**) demonstrates location of BrdU (28-day-old bromodeoxyuridine labeled) cells in close proximity to PECAM-1 cells in the prefrontal cortex of the adult rat brain, and additional phenotypic analysis of BrdU cells shows that they develop into premyelinating oligodendrocytes [26]. The later part of the review discusses the relationship between oligodendrocytes and PECAM-1 and their potential role in alcohol use disorder (AUD).

Adhesion molecules allow the interaction between immune cells and endothelium. The adhesion molecules belong to the mucin, selectin, cadherin, integrin and Ig superfamily [39,40]. PECAM-1 is a type I transmembrane adhesion protein of 130 kDa, which belongs to a subgroup of the Ig superfamily, characterized by the presence of immunoreceptor tyrosine-based inhibitory motifs. PECAM-1 is encoded by a 65-kb gene allocated in the long arm of chromosome 17 in humans [41] and chromosome 10 in rats [42], and the region driving its transcription has been identified as a TATA-less promoter containing relevant EGR-1 and GATA-2 cis-regulatory elements [43,44]. Structurally, the extracellular domain of PECAM-1 contains 6-Ig homology domains capable of both homophilic and heterophilic binding [45,46]. PECAM-1 has a single transmembrane domain, and a cytoplasmic domain characterized by its dual immunoreceptor tyrosine-based inhibition motif (ITIMs), and eight exons that are subjected to alternative splicing [46,47,48]. Detailed discussions about the significance of the structural domains have been provided elsewhere [37,45,46,49,50,51,52,53,54]. Here structural components of PECAM-1 are presented briefly in the context of its function in physiological and pathological conditions. PECAM-1 is also identified as cluster of differentiation 31 (CD31) as well as the endothelial cell junctional protein. PECAM-1 is a glycosylated adhesion molecule abundantly expressed on the endothelial cells (blood vessels and capillaries) and (to a lesser extent) on hematopoietic cells (platelets, monocytes, neurtophils) and immune cells (B cells and T cells) [37,46,47,55]. Note, PECAM-1 is not known to be expressed on neurons or glia [56]; in the brain PECAM-1 is exclusively expressed on cerebral endothelial cells that make up the blood-brain barrier (Figure 1). PECAM-1 has several functions that are critical in healthy physiology as well as in recovery following injury or other pathological conditions, and the following sections will elaborate on the function of PECAM-1 on endothelial cells in the context of inflammation and apoptosis [46,54].

### 2.2. Function of PECAM-1 on the Endothelial Cells

**Figure 2:**
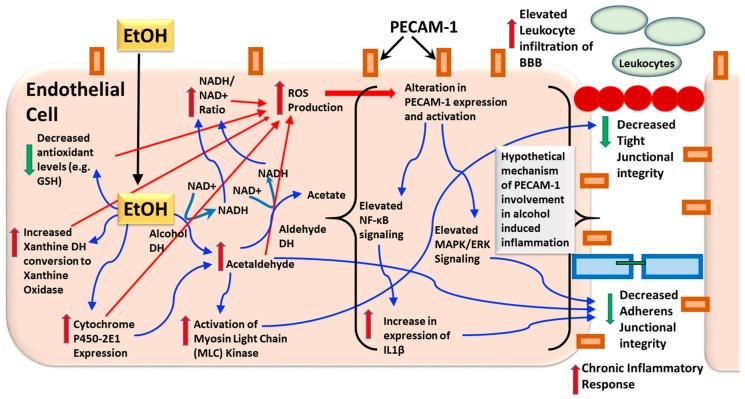
Schematic of an endothelial cell in the periphery with expression of PECAM-1 and alterations in signaling and expression of proteins by alcohol (EtOH) experience. Blue arrows indicate direct actions of EtOH in the cell, red arrows indicate secondary actions of EtOH in the cell. Hypothetical involvement of PECAM-1 in alcohol-induced inflammatory signals is indicated in brackets in the endothelial cell.

PECAM-1 is expressed abundantly on the cell membrane and in the intercellular junction between endothelial cells (Figure 2); this localization is mediated largely by PECAM-1/PECAM-1 homophilic binding via the first two Ig-domains [46,55,57]. Here PECAM-1 functions as a biosensor that modulates vascular permeability in response to variations in blood-flow or to osmolarity changes [55,58,59,60]. Homophilic binding between PECAM-1 receptors on endothelial cells and on leukocytes is involved in transendothelial migration of leukocytes, a process involving transient increase in vascular permeability [55,61,62,63,64]. Of note, PECAM-1 itself is not a component of the tight junctions (claudins and occludins), or the adheren junctions (vascular endothelial (VE)-cadherin and catenins) which bind to the actin cytoskeleton of endothelial cells to physically regulate vascular permeability [46,55,65]. In contrast, PECAM-1 facilitates the activation of β-catenin, which then associates with and supports the function of VE-cadherin [46,66]. Importantly, PECAM-1 deficient mice exhibit no vascular abnormalities under normal conditions [65]. However, vascular resilience in the presence of a physical stressor or an inflammatory challenge, as well as vascular recovery after a barrier breach are severely impaired in PECAM-1 deficient mice [36,65,67,68,69,70]. Taken together, PECAM-1 regulates vascular permeability (without direct interaction with junction proteins) by modulating the structure and function of tight junction and adherens junction proteins, in response to inflammation, injury or mechanical stress on vasculature.

PECAM-1 signaling is typically initiated by phosphorylation of the ITIMs by Csk and Src families of protein tyrosine kinases (PTKs), followed by the recruitment of proteins that contain the Src homology 2 (SH-2) domain (example, SH-2 domain-containing protein-tyrosine phosphatase or SHP-2) [49,50,53,54]. This binding activates mitogen-activated protein kinase/extracellular-signal-regulated kinase (MAPK/ERK) pathway to allow increased vascular permeability, and to enable endothelial cell migration that is critical for angiogenesis [27,45,55]. As mentioned above, the cytoplasmic domain of PECAM-1 is subjected to alternative splicing and splice variants of PECAM-1 are hypothesized to be expressed in tissue selective and developmentally specific manner [27,47]. Of interest, splice variants lacking a particular exon (exon 14) are incapable of binding SHP-2 rendering the cells incapable of triggering the above pathway [27,71], and as such, are more predominant in mature vasculature [72]. Overexpression of PECAM-1, observed during recovery from ischemic damage [73,74] is hypothesized to transiently restore the angiogenic phenotype by “isoform switching” to exon 14 containing PECAM-1.

### 2.3. Role in Inflammatory Responses

The role of PECAM-1 in inflammation is multifaceted, where studies have supported the anti-inflammatory properties and pro-inflammatory characteristics of the molecule. The anti-inflammatory roles of PECAM-1 include inhibition of leukocyte activation, dampening cytokine production during inflammation, and providing resilience to endothelial barrier cells against inflammatory challenges (for review, [64]). Furthermore, endothelial PECAM-1 (and not leukocyte PECAM-1) was found to be protective against excessive inflammation in an animal model of multiple sclerosis [68]. These anti-inflammatory properties are dependent on PECAM-1 signaling mechanisms including, ITIM-phosphorylation and recruitment of SH-2 domain containing binding partners [68,70,75], enhanced phosphorylation of signal transducer and activator of transcription-3 (STAT3) [70] and decreased translocation of nuclear factor kappa B (NF-kB) in the nuclei of endothelial cells [76]. These findings suggest that the anti-inflammatory roles of PECAM-1 are limited to certain physiological states, and conditions that do not involve severe oxidative stress and vascular inflammation.

The most well characterized pro-inflammatory role of endothelial PECAM-1 is the contribution of PECAM-1 to trans-endothelial migration of white blood cells (monocytes and neutrophils; [77,78]). This function is dependent on PECAM-1 homophilic and hetereophilic binding with the leukocytes, and is not contingent of ITIM-mediated signaling [52,64,79]. Interestingly, this response is triggered by interleukin-1β (IL1β), but not other pro-inflammatory molecules such as tumor necrotic factor-α (TNFα) or other chemokines (Figure 2; [37,61,62,80,81]. With respect to the inflammatory transcription factor NF-kB, several interesting lines of evidence demonstrate that PECAM-1 could enhance the expression of NF-kB, and NF-kB could increase the transcription of PECAM-1. For example, it is important to note that PECAM-1 regulation of NF-kB occurs under conditions of oxidative stress-induced inflammation in rodent models of blood-flow restriction and in ischemia/reperfusion injury via tyrosine phosphorylation [58,82], and does not occur in in vitro conditions that involve sheer overexpression of PECAM-1 [83]. Mechanistic studies show that NF-kB mediated pro-inflammatory effects of PECAM-1 is hypothesized to be mediated via phosphoinositol-3-kinase/Akt (PI3K/Akt) signaling pathway [58]. With respect to NF-kB regulation of PECAM-1, molecular studies have identified two consensus sites for NF-kB within the promotor region of PECAM-1 gene [44], and few studies have demonstrated functional relevance for this interaction, where NF-kB regulates transcriptional activity of PECAM-1 [84], and vascular inflammation mediated by PECAM-1 is dependent on NF-kB activity [85]. Given the extensive evidence that NF-kB is implicated in the neuroinflammatory responses in AUDs [34,86] and the limited evidence that PECAM-1 could be involved in the neuroinflammatory responses in AUD [26,87], mechanistic studies understanding the relationship between PECAM-1 and NF-kB in the context of AUDs is an important future pursuit.

### 2.4. Role in Apoptosis

Cytoprotection of endothelial cells is another critical function of PECAM-1. In fact, the same N-glycosylation of the Ig-domains that mediate localization of PECAM-1 at the endothelial cell-cell junction, are also implicated in anti-apoptotic signaling that enhances endothelial cell survival in culture [51,52,57]. Apoptotic cell death in endothelia as well as in neurons and glia can be triggered by an extrinsic pathway or an intrinsic apoptotic pathway, or by a cross-talk of the two pathways [88,89,90,91]. Briefly, the extrinsic pathway is triggered by the activation of ‘death receptors’ that cause the activation of caspase 8 enzymes that ultimately cleaves and activates the effector caspases such as caspase 3. The intrinsic pathway is mitochondria-dependent, whereby cellular insults (via free-radicals, etc.) lead to activation of pro-apoptotic proteins of the B-cell lymphoma 2 family (Bcl2 family, for example Bax and Bak), releasing cytochrome C from the mitochondria, and that finally converge onto activation of caspase 3. PECAM-1 exerts its anti-apoptotic function by inhibiting components of both the intrinsic and the extrinsic pathways via activation of PI3K/Akt signaling pathway [54,92]. Specifically, PECAM-1 mediated activation of PI3K/Akt pathway upregulates NF-kB-mediated transcription to facilitate angiogenesis, cell survival/growth and recovery of endothelial cell barrier [58,64,82]. In this manner, PECAM-1 is able to mediate protection against extrinsic apoptotic pathways that are instigated TNFα signaling, or as a consequence of endothelial barrier damage [71,90,92,93]. PECAM-1 dependent PI3K/Akt pathway activation has been shown to upregulate expression of anti-apoptotic Bcl-2 family proteins (including Bcl-xL and Bcl-2), thus preventing the intrinsic apoptosis [71,92]. Additionally, PECAM-1 may confer its anti-apoptotic effects indirectly by modulating STAT-3 activity or by modulating intracellular calcium signaling [35,70,94,95]. Taken together, PECAM-1 signaling is capable of cytoprotection of the endothelial cells by supporting anti-apoptotic pathways. This has been shown to promote tumorigenesis, especially by allowing vascularization of solid tumor [92]. Whether, such properties enable PECAM-1 to support non-tumorigenic cell proliferation in non-endothelial brain cells is a topic of interest, especially if such processes may help in the recovery of brain cells.

### 2.5. Role in AUD

**Figure 3:**
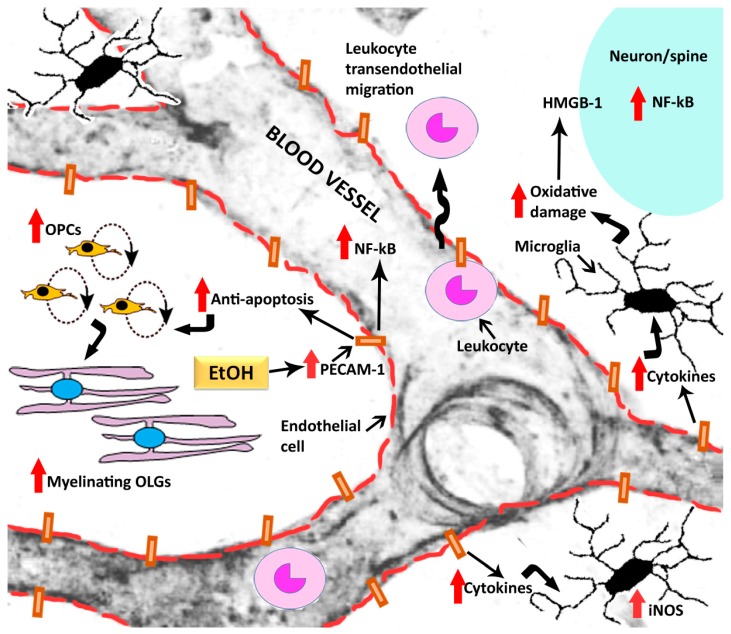
Schematic of blood vessel in the brain and hypothetical mechanisms of alcohol (EtOH) in the brain that are associated with alcohol-induced enhanced expression of PECAM-1. Attention is paid to include increases in expression of nuclear factor kappa b (NF-kB), activation of microglia and transendothelial migration of leukocytes as inflammatory responses post alcohol experience. Open headed arrows indicate the cell type; closed headed arrows indicate a mechanism followed by direction of effect; red thick arrows indicate increases in protein expression or effect on the signaling mechanism. OPCs, oligodendrocyte progenitor cells; OLGs, oligodendrocytes; HMGB-1, high mobility group box 1.

Very few studies have directly investigated PECAM-1 in relation to alcohol, particularly as it pertains to the ethanol toxicity and addiction. Most of PECAM-1’s role discussed here has been inferred from PECAM-1 related signaling partners implicated in alcohol’s effects on the blood-brain barrier. As discussed in the sections above, much of the endothelial damage produced by chronic or high doses of alcohol are mediated by triggering pro-inflammatory processes [17,20], and have been presented as such in a simple schematic (Figure 3). Briefly, alcohol readily diffuses across the lipid bilayer of blood brain barrier and is metabolized into acetaldehyde by enzymes such as cytochrome P450-2E1 and alcohol dehydrogenase [20]. Acetaldehyde can directly catalyze endothelial cadherin-catenin complexes and occludins, which are critical components of the adherens junctions and tight junctions, respectively, [66,96] to affect blood-brain barrier disruption. Alternatively, acetaldehyde can facilitate the production of ROS through the activation of enzymes like NADPH oxidase and nitric oxide synthase (NOS) in all types of cell (neurons, glia, and brain endothelial cells) [19,97,98]. High doses of alcohol also activates other stress related pathways such as xanthine oxidase, and enhanced xanthine oxidase by alcohol could be facilitated via alcohol-induced glutamate toxicity [99]. Enhanced xanthine oxidase and other cellular mechanism (e.g., increased NADH/NAD+ ratio, glutathione (GSH) depletion, enhanced cytrochrome-P450 expression) that contribute to enhanced ROS could contribute to PECAM-1 activation (Figure 2). For example, ROS and NOS can trigger prolonged upregulation of NF-kB signaling, thereby enhancing inflammatory and immune responses that further contribute to the neuropathology associated with alcohol addiction [13,17,19]. These mechanisms may be linked back to the transendothelial migration of leukocytes promoted by PECAM-1 [38] or to modulation of VE-cadherin (in adherens junctions) by PECAM-1 [61,78]. Alternately, the above mechanism may act synergistically or antagonistically with PECAM-1-mediated inflammatory signaling pathways. Therefore, future mechanistic studies are needed to determine the role of PECAM-1 in mediating neuroinflammatory responses in AUDs.

Given the angiogenic and anti-apoptotic roles of endothelial PECAM-1, recovery following alcohol toxicity during abstinence may be closely linked to PECAM-1. For example, acute ethanol was shown to inhibit angiogenesis during wound healing, which may be associated with the pro-apoptotic effects of ethanol via the Bax/Bcl pathways [100,101]. Note, these acute intoxication related damages may be unavoidable as the mature brain typically expresses PECAM-1 isoforms lacking exon 14, which is essential for its anti-apoptotic function [47]. As observed during recovery from ischemic damage, isoform switching to reinstate the angiogenic, anti-apoptotic isoform may occur to enable repair and remodeling of the damaged blood-brain barrier [73,74]. Therefore, similar processes may be recruited during prolonged alcohol abstinence to enable the recovery of cerebral endothelium as well as other neural cells damaged by alcohol toxicity [71,92]. Evidence supporting such a time-line was provided by our laboratory, where increased PECAM-1 expression was observed in the rodent neocortex during protracted abstinence from chronic ethanol administration [87]. Whether such processes contribute to the recovery of neuronal function remains controversial. The PECAM-1 upregulation during protracted alcohol abstinence was associated with suppressed neuronal activation and enhanced oligodendroglial proliferation in the prefrontal cortex (a brain region implicated in addiction) as well as with maladaptive high-levels of ethanol seeking [87,102]. Recent data also found that the blood-brain barrier integrity, as measured by expression of endothelial barrier antigen, was not fully recuperated at the same time-point [26]. In an in vivo experiment evaluating chronic ethanol administration in alcohol preferring rats, brain endothelial cells of ethanol-drinking rodents exhibited permanent increase in blood-brain barrier permeability post endotoxin challenge (i.e., lipopolysaccharide, LPS) [103]. This enhanced permeability was shown to correlate with decreased transcriptional expression of critical tight junction proteins, and paradoxically, was associated with neuroprotection against LPS mediated apoptosis. Others report that withdrawal from chronic ethanol usage is a dynamic state, during which there is significant upregulation of pro-inflammatory signals, including elevated cytokine production and enhanced transcriptional expression of mediators of toll-like receptor (TLR)-4-dependent neuroimmune signaling [104,105,106]. Taken together, the upregulated PECAM-1 expression observed during protracted abstinence may explain, in part, the continued inflammation, suppressed apoptosis and retarded recovery of the endothelial barrier [50], although further isoform analyses and mechanistic studies are needed to test this hypothesis. Furthermore, effect of PECAM-1 modulation on suppression of neuronal activation in response to alcohol related cues is a topic of interest as a potential therapeutic strategy for addressing the increased risk for relapse in AUD.

## 3. Oligodendrocytes and Oligodendrogenesis

In higher order mammals, almost all neuronal cells at birth are incapable of proliferation, with the notable exceptions of neuronal progenitors in the subventricular zone of the olfactory bulb and the subgranular zone of the hippocampal dentate gyrus [107]. In contrast, de novo oligodendrocytes (OLGs), capable of maturing into myelinating OLGs, are produced throughout the lifetime of adult mammals via a process known as oligodendrogenesis [108,109,110,111]. A detailed review of specific markers for the various stages of oligodendrogenesis can be found elsewhere [112,113,114]. The best understood and widely utilized marker for OLGs is Oligodendrocyte transcription factor (OLIG2), a helix-loop-helix transcription factor that is expressed through all the stages of oligodendroglial maturation [115,116,117,118,119,120]. Knockdown and upregulation of OLIG2 led to a decrease and increase, respectively of oligodendrocyte progenitor cells (OPCs) that are identified using the neuron-glial 2 (NG2) marker [121,122]. Interestingly, although OPCs were found to be multipotent in vitro, overwhelming evidence in vivo suggests that the neurogenic potential of OPCs is very limited and OPCs predominantly differentiate into OLGs [110,123,124,125,126]. Additionally, neural stem cells from the subventricular zone of the olfactory bulb are considered to be a continuous source of OPCs in the adult brain [122,127,128,129]. Notably, OPCs are functional and contribute to several neuron-glial interactions. For example, some OPCs have voltage gated Na+ channels which generate action potentials and receive electrical inputs from neurons [130]. Additionally, OPCs express receptors for several key neurotransmitters and neuromodulators, including but not limited to glutamate, γ-aminobutyric acid and dopamine [131,132,133,134,135]. Under healthy, physiological conditions, electrical and neurochemical inputs from neurons are shown to modulate oligodendrogenesis and myelination [136,137,138,139]. Furthermore, growth factors released from neurons are also involved in modulating proliferation, migration, differentiation and myelination by OLGs [139,140,141,142], suggesting that neuronal plasticity modulates OLG and OPC plasticity. In addition to the neuroplasticity effects on OPCs and OLGs, several recent studies support the involvement of the cerebrovascular system in providing trophic support to the maintenance of OPCs and OLGs [143]. For example, interactions between OLGs, myelin, endothelial cells and neuroinflammatory proteins have been demonstrated in models of brain injury, including, stroke, ischemia and AUD [87,144,145]. Furthermore, OPCs are positioned at close proximity to endothelial cells in the adult brain (Figure 1h; [26], and proliferation and survival of OPCs is regulated by vascular endothelial cells, such that increase in endothelial response enhances OPC proliferation and survival [146]. Therefore, a deeper understanding of the mechanisms of endothelial-OLG and endothelial-OPC trophic coupling may lead to new therapeutic approaches for myelin- and vascular inflammation-related diseases, such as stroke and AUDs.

### Role of OLGs and OPCs in AUD

While loss of white matter itself is a critical part of neuropathology of AUDs [147], this section of the review is focused on OLGs and OPCs in the context of alcohol-induced neuropathology. We recently reviewed the potential role for impaired oligodendroglial proliferation and abnormal maturation in addictive disorders [111]. For example, OLG homeostasis was disrupted by both psychostimulants like methamphetamine as well as sedative hypnotics, like alcohol [126,148,149,150]. With respect to alcohol, proliferation and survival of OPCs in the prefrontal cortex (PFC) were suppressed by a chronic intermittent ethanol exposure paradigm (CIE) [149,150], a widely established model of moderate to severe AUD. Furthermore, this suppression of proliferation was found to be transient, and a proliferative burst was observed 72 h post CIE [87,102]. These hyperproliferating OPCs in the PFC survived into protracted abstinence [87,102], differentiated into OLIG2-positive OLGs [87,111,126,148,150], and matured into myelinating OLGs [87]. One may suggest that the enhanced oligodendrogenesis in the PFC is a compensatory (neuroprotective) mechanism that contributes to the recovery of the cortical tissue lost due to AUD [151]. Support for this hypothesis is provided by the observation that myelin levels in the PFC are inversely linked to neuronal activity in the PFC and to stress-induced relapse vulnerability [152]. Therefore, increase in myelin basic protein observed during protracted abstinence from CIE may reduce propensity for relapse [153]. Contradicting this hypothesis, we recently reported that increased myelin basic protein and oligodendeogenesis during protracted abstinence from alcohol were associated with decreased neuronal activation (FOS) in the PFC, and these changes were associated with increased relapse to cue-mediated ethanol seeking [87]. Taken together these results suggest that the relation between OLG, myelination and behavior is complex and several other factors may weigh in on accurate determination of relapse risk. In that regard, we will now discuss the possible interaction of PECAM-1 in this relation between OLG and AUD.

## 4. Interaction of Oligodendrogenesis and PECAM-1 in AUD

Very few studies have investigated the relationship between cerebral endothelial cells and oligodendrogenesis in pathophysiology and one study as it pertains to AUDs [26]. For example, rodent model of binge ethanol exposure demonstrate that ethanol-induced cytokine responses disrupts myelin associated proteins [154], which is associated with reduced OPCs and with cognitive deficits [149,155,156]. Given the role of PECAM-1 in mediating neuroinflammatory response by endothelial cells [30,64,157,158], it is fairly intuitive that PECAM-1 may be involved in these effects [106,159].

In this context, during prolonged abstinence from alcohol in an animal model of AUD, higher PECAM-1 expression was associated with higher oligodendrogenesis and higher reinstatement of cue-mediated alcohol seeking [87]. Access to running wheel reversed these effects such that normalized PECAM-1 expression and oligodendrogenesis were associated with lower reinstatement of ethanol seeking [87]. Notably, in the animal model of AUD and not in rats modeling low levels of social-drinking like behaviors, increased oligodendrogenesis was observed juxtaposed with PECAM-1 labeled cells [26]. These and others studies report that OPCs are located close to cerebral endothelia, and under pathological conditions may contribute towards weakening of the endothelial barrier in a paracrine manner [160]. In contrast to the effects of alcohol toxicity during intoxication and prolonged abstinence, exercise and access to running wheels have been shown to reduce NG2-OPCs and increase differentiation of OPCs [126,161]. Exercise helped protect blood-brain barrier and protect critical neuronal function in other diseases, such as cerebral ischemia, multiple sclerosis as well as addiction [67,162,163,164]. Taken together, exercise may exert opposite effects on OPCs depending on the pathological history of the subject, a phenomenon that is reminiscent of the effects of cytokines IL-1β and TNF-α on oligodendrogenesis [109,165]. These neuroinflammatory and protective effects may be mediated by PECAM-1-dependent leukocyte transendothelial migration as well as by PECAM-1’s effects on apoptotic mechanisms [46,78,82,92,166]. As mentioned previously, all OPCs and myelination are not identical, and by extrapolation beneficial. Future studies should further investigate potential differences in OPCs and OLGs generated during physiological and pathological conditions, and whether history of drug-induced neurotoxicity alter the functional properties of these cells.

## 5. Conclusions

Chronic ethanol exposure induces oxidative stress and neuroinflammation, in part mediated by endothelial PECAM-1, which may contribute to blood-brain barrier damage, and reduced oligodendrogenesis and demyelination and cognitive deficits. Abstinence from alcohol may not necessarily reverse the blood-brain barrier damage or reduce risk of relapse. However, oligodendrogenesis and expression of myelin-related proteins are increased and some aspects of cognitive deficits are ameliorated. These effects along with upregulation of PECAM-1 could be physiological mechanisms of recovery from ethanol-induced oxidative damage or alternatively, be evidence for vascular/endothelial inflammation driving maladaptive plasticity.
